# Simultaneous Enhancement of Photocatalytic Bactericidal Activity and Strength Properties of Acrylonitrile-Butadiene-Styrene Plastic Via a Facile Preparation with Silane/TiO_2_

**DOI:** 10.3390/polym12040917

**Published:** 2020-04-16

**Authors:** Kunlanan Kiatkittipong, Jun Wei Lim, Chin Kui Cheng, Worapon Kiatkittipong, Suttichai Assabumrungrat

**Affiliations:** 1 Department of Chemical Engineering, Faculty of Engineering, King Mongkut’s Institute of Technology Ladkrabang, Bangkok 10520, Thailand; 2 Department of Fundamental and Applied Sciences, HICoE-Centre for Biofuel and Biochemical Research, Institute of Self-Sustainable Building, Universiti Teknologi PETRONAS, Seri Iskandar 32610, Malaysia; 3 Faculty of Chemical and Natural Resources Engineering, Universiti Malaysia Pahang, Pahang 26300, Malaysia; 4 Department of Chemical Engineering, Faculty of Engineering and Industrial Technology, Silpakorn University, Nakhon Pathom 73000, Thailand; 5 Center of Excellence in Catalysis and Catalytic Reaction Engineering, Department of Chemical Engineering, Faculty of Engineering, Chulalongkorn University, Bangkok 10330, Thailand

**Keywords:** titania photocatalyst disinfection, photocatalytic degradation, aptes treatment, photocatalytic inactivation, photokilling

## Abstract

This work aims to enhance the photocatalytic antibacterial performance of plastics according to the JIS Z 2801:2010 standard, and to determine their mechanical properties by studying: (i) the influence of calcination on titanium dioxide (TiO_2_); (ii) modification with different TiO_2_ concentrations, and; (iii) the effect of silane as a coupling agent. Acrylonitrile-butadiene-styrene plastics (ABS) and *Escherichia coli* (*E. coli*) were chosen as the model plastic and bacteria, respectively. The 500 °C calcined TiO_2_ successfully provided the best photoantibacterial activity, with an approximately 62% decrease of *E. coli* colony counts following 30 min of exposure. Heat treatment improved the crystallinity of anatase TiO_2_, resulting in low electron-hole recombination, while effectively adsorbing reactants on the surface. ABS with 500 °C-calcined TiO_2_ at the concentration of 1 wt % gave rise to the highest performance due to the improved distribution of TiO_2_. At this point, blending silane coupling agent could further improve the efficacy of photoantibacterial activity up to 75% due to greater interactions with the polymer matrix. Moreover, it could promote a 1.6-fold increase of yield strength via increased adherent bonding between TiO_2_ and the ABS matrix. Excellent photocatalytic and material stability can be achieved, with constant photocatalytic efficiency remaining for up to five reuse cycles without loss in the yield strength.

## 1. Introduction

Thermoplastic polymers are widely used for appliances such as sanitary ware, medical appliances, furniture and children’s toys due to their favorable properties, such as excellent impact resistance, good machinability and excellent aesthetic qualities [1]. Durable plastics with a useful life of three years or more, such as Acrylonitrile-Butadiene-Styrene (ABS) plastics, could be potentially colonized by myriad microorganisms. Therefore, one of the biggest problems these plastics pose is microorganism contamination, including viruses, fungi and bacteria, which are harmful to the environment, hazardous to humans, and are difficult to disinfect. The generated reactive oxygen species (ROS) play a crucial role in bacterial disinfection. ROS can be generated by light irradiation or, more recently, by harnessing the power of microwaves using a conventional microwave oven [2,3].

Photocatalysis have attracted considerable attention in solving bacterial contamination as a clean, energy-efficient, low-cost and environmentally friendly technology. Photocatalytic bacterial inactivation relies upon the generation of highly reactive short-lived hydroxyl radicals and reactive oxygen that efficiently damage the cell membranes of microorganisms. TiO_2_ is widely used for photocatalytic applications due to its high stability, low cost and non-toxicity [4,5]. For example, Ti-O nanostructures synthesized from TiO_2_ in alkaline hydrothermal conditions have enhance photocatalytic reaction rates and sedimentability [6]. Lee and Chang reviewed composite photocatalysts widely used for degradation of hazardous chemicals, antibacterial, and photocatalytic hydrogen production [7]. Polymeric membranes with TiO_2_ showed excellent degradation of pollutants in water due to active sites of TiO_2_ available for interacting with photons [8]. Moreover, in-situ-formed granular TiO_2_ on polyvinylidene fluoride (PVDF) fiber with graphitic carbon nitride exhibited excellent self-cleaning properties [9]. 

Coating TiO_2_ on polymers such as polypropylene [10,11], polyethylene terephthalate [12], polycarbonate [13], and polystyrene [14] has been proposed to reduce the abundance of *Escherichia coli* (*E. coli*) and other harmful microrganisms. The properties of TiO_2_ such as crystallinity, surface defects, and particle size may affect its photocatalytic activities. However, potential flaking-off of TiO_2_ is an issue when the polymer is used many times. This may increase the risk to human health and result in environmental hazards. Apart from the effect on microorganisms, awareness of the mechanical properties of thermoplastic polymers is another concern. Many studies have reported that the filler adhesion and dispersion on the matrix of polymers were the main factors [15].

There is a body of literature on the effects of TiO_2_ on physical and chemical characteristics of ABS composites. Adding commercially surface-treated TiO_2_ pigment to ABS shows some optical benefits; i.e., doing so can impart opacity and whiten the ABS polymer as indicated by an increasing whiteness index and decreasing yellowness index. Impact strength of TiO_2_-pigmented ABS improved with a small loading of TiO_2_ pigment [16]. The friction and wear properties of TiO_2_-ABS were investigated by varying the TiO_2_ content, normal load, and sliding speed. With optimization of these parameters, the friction and wear properties of TiO_2_-ABS could be improved [17]. Recently, TiO_2_-ABS nanocomposite filaments have been produced and applied in 3D printing. TiO_2_-ABS with 5 and 10 wt % loading possess higher breaking point stress thresholds than those printed from the pure ABS polymer. A 10% TiO_2_-ABS preparation exhibited a marked photocatalytic activity leading to dye degradation, as observed by decreasing fluorescence emission spectra of rhodamine 6G by approx. 22% over 4 h of light exposure [18].

Although many studies have demonstrated the development of thermoplastic polymers in terms of their mechanical properties or in relation to microorganism suppression, there has been limited assessment of the antibacterial role of TiO_2_, and its effects on the strength of plastics. In particular, there is no previous study available on ABS plastic—a material that provides effective and quality products for various applications. The most relevant literature is our preliminary study of TiO_2_/ABS on photoantibacterial activity, without functionality or interfacial affinity modification, which resulted in unimpressive photoactivity [19]. Moreover, the impact of calcination and fillers on TiO_2_ properties, and its potential photoantibacterial effect, have not been investigated of late. This study focuses on improving photoantibacterial performance following the standard JIS Z 2801, which covers the ability of plastic surfaces to inhibit the growth of microorganisms. We also investigate strength properties (per standard ASTM D639 Type I) of ABS plastic with modified TiO_2_. The role of calcination temperature, concentration and silane-coupling agent is also assessed.

## 2. Materials and Methods 

### 2.1. Reagents

TiO_2_ (anatase 100%), acrylonitrile-butadiene-styrene (ABS Resin, PA—717C Grade), 3-aminopropyltriethoxysilane (APTES) (Sigma–Aldrich, St. Louis, MI, USA), stock phosphate buffer solution (PBS) (Sigma–Aldrich), plate count agar (PCA), tryptic soy broth (TSB), peptone (J.T.Baker) were used. 

### 2.2. Calcination of TiO_2_ Powder

Anatase TiO_2_ particles were preheated in an oven at 90 °C for 2 h, and then calcined at temperatures of 300, 500 and 800 °C for 2 h with a ramping rate of 5 °C min^−1^.

### 2.3. Surface Modification of TiO_2_ with Silane

A 0.5 g mass of calcined TiO_2_ was dispersed in 50 cm^3^ of 2.5% *v*/*v* ethanol. Then, 0.15 g of the silane-coupling agents (APTES) at standard conditions were added in the dispersion, and stirred for 45 min. The resulting slurry was centrifuged and dried in an oven for 24 h at 80 °C

### 2.4. Preparation of TiO_2_/ABS Compositions

Prior to blending polymers, ABS plastic was dried in an oven at 90 °C for 2 h to remove moisture. The TiO_2_ or calcined TiO_2_ particles (concentration of 0.5, 1 and 2 wt %) or calcined TiO_2_ treated with silane, and ABS plastic were loaded into a twin-screw extruder, and then mixed well in an internal mixer at 250 °C with speed round mixing at 60 rpm for 6 min. The melting material was put in the mold (5 × 5 cm^2^) by using a compression molding at a temperature of 250 °C and pressure of 125 kg cm^−2^ for 5 min, and rapidly cooled for 5 min. The resulting TiO_2_/ABS compositions were air-cooled at room temperature before being tested.

### 2.5. E. coli Bacteria Preparation and Photoantibacterial Activity

Photoantibacterial activity and efficacy assessments followed the standard JIS Z 2801 (The test for antibacterial activity and efficacy on surfaces of antibacterial products). A colony of *E. coli* bacteria was transferred into TSB solution, and then incubated at 32 ± 0.5 °C for 24 h. The bacterial suspensions were diluted into 2.5 × 10^8^ cfu cm^−3^, dropped on TiO_2_/ABS workpieces, and covered with a polyethylene (PE) film size 4 × 4 cm^2^. The sample was illuminated by UVC light (15 W) for 30 min. The bacterial population was determined by plated serial dilution, and plates were incubated at 32 ± 0.5 °C for 24 h. All photoantibacterial activity experiments were performed with three replicates (independent experiments), and the data were represented as the average mean ± SD (error bar).

### 2.6. Sample Characterization 

Crystal and structural characteristics of the products and crystallinity were investigated using powder X-ray diffraction (XRD) system with monochromatized Cu_kα_ radiation (λ = 1.5406 Å). Full width at half maximum (FWHM) derived from XRD patterns at 2θ = 25° indicated the degree of crystallinity. Sample morphology was investigated by a scanning electron microscope, and the surface area and pore size distribution were determined by N_2_ adsorption. 

### 2.7. Mechanical Tensile Strength

The workpieces were tested using a universal testing machine according to the guidelines set in ASTM D639 Type I (Standard Test Method for Tensile Properties of Plastics). All experiments were performed with three replicates, and the data were represented as the average mean ± SD (error bar).

## 3. Results and Discussion

### 3.1. Effect of Calcination Temperatures on TiO_2_

**Figure 1:**
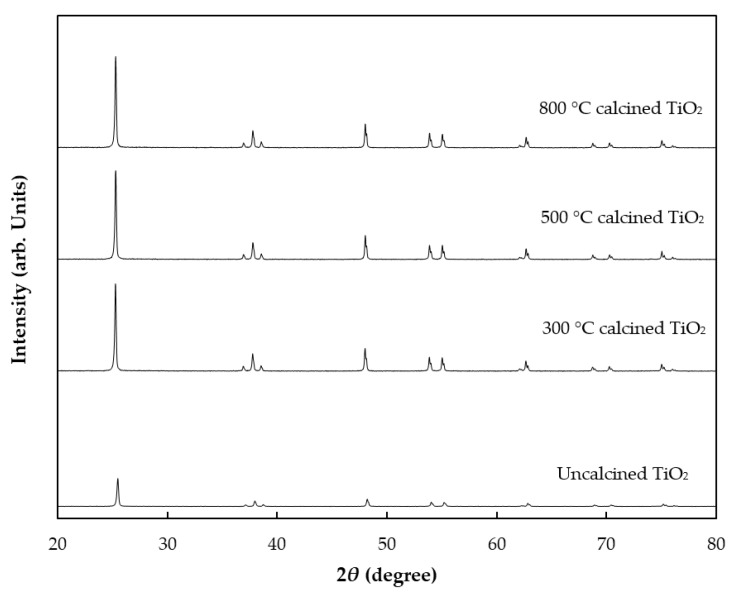
XRD patterns of uncalcined TiO_2_ (bottom) and TiO_2_ calcined at different temperatures.

The crystal structure of TiO_2_ was investigated by XRD analysis as shown in Figure 1 and corresponding crystallite size was calculated by using the Debye–Scherrer formula. The XRD pattern demonstrated that TiO_2_ structures were influenced by calcination temperatures. The initial TiO_2_ results included peaks at 2θ = 25.4°, 37.8°, 48°, 54.5° and 62.8°, corresponding to the anatase phase of TiO_2_ with the FWHM value of 0.5 and particle size of 6.12 nm. No peaks caused by impurities were observed. TiO_2_ calcined at 300 °C showed that the intensity of crystallinity of anatase increased, with a lower FWHM value of 0.41 and particle size of 7.08 nm. Further increases in temperature to 500 and 800 °C increased crystallinity of anatase, as shown by recorded FWHM values of 0.27 and 0.08, respectively. Therefore, crystalline structure was slightly developed by increasing the calcination temperatures. The particle sizes of 500 and 800 °C calcined TiO_2_ increased slightly to 7.13 and 7.15 nm, respectively.

**Figure 2:**
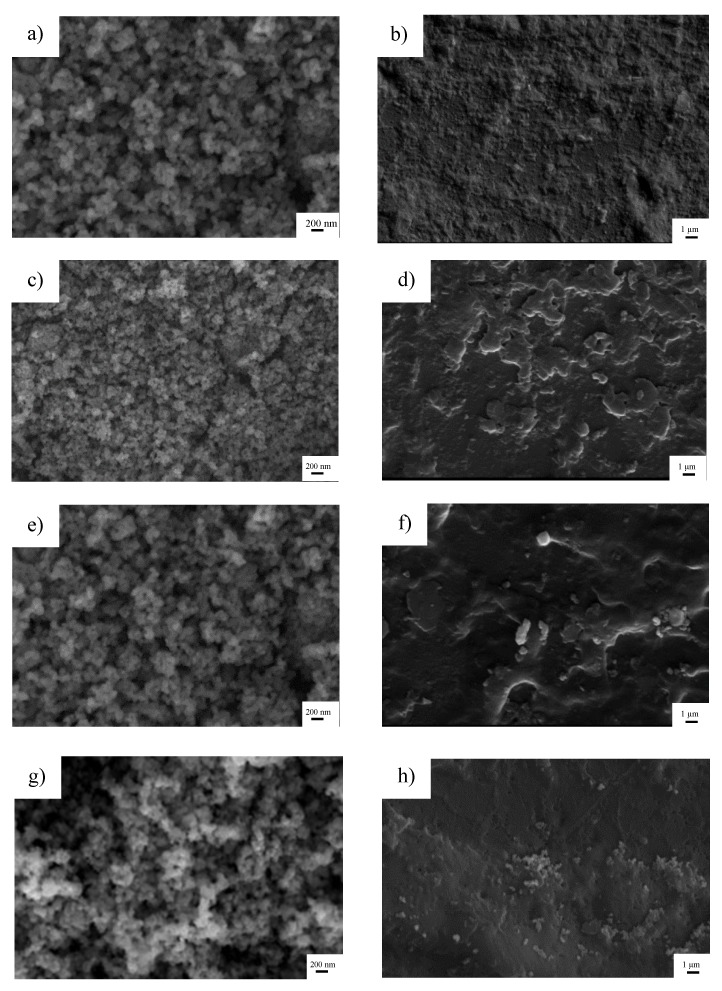
SEM images of (**a**) TiO_2_, (**b**) TiO_2_/ABS, (**c**) 300 °C calcined TiO_2_, (**d**) 300 °C calcined TiO_2_/ABS, (**e**) 500 °C calcined TiO_2_, (**f**) 500 °C calcined TiO_2_/ABS, (**g**) 800 °C calcined TiO_2_, (**h**) 800 °C calcined TiO_2_/ABS.

The initial surface area of TiO_2_ was 10 m^2^ g^−1^. At 300 °C, the specific surface area was reduced to 9 m^2^ g ^−1^. A continuous decrease in surface area with the rise of calcination temperature was observed in TiO_2_ calcined at 500 and 800 °C, with respective surface areas of 7 and 4 m^2^ g^−1^. The influence of calcination temperature on morphology was investigated by SEM imaging as presented in Figure 2. Initially, the TiO_2_ particles had a diameter of approximately 200 nm as shown in Figure 2a. When mixing TiO_2_ particles with ABS plastic, the particles appeared to be embedded in the ABS plastic, resulting in the rough surface, as observed in Figure 2b. Calcining at 300 °C led to structural aggregation of TiO_2_ particles (Figure 2c), whereby the surface of ABS plastic contained assemblies of particles in some areas (Figure 2d). At 500 °C, it was also observed that a continuous aggregation in TiO_2_ particles occurred (Figure 2e) and these were visibly assembled on the ABS surface (Figure 2f). When the temperature was increased to 800 °C, the TiO_2_ particles became obviously aggregating as seen in Figure 2g. Calcination of TiO_2_ results in partial or total collapse of the structure, decreasing of surface area, and the appearance of particle agglomerations. This resulted in poorly dispersed distribution of TiO_2_ particles on ABS plastic as shown in Figure 2h. The higher degree of agglomeration was ordered as follows: 800 °C-TiO_2_/ABS > 500 °C-TiO_2_/ABS > 300 °C-TiO_2_/ABS > uncalcined-TiO_2_/ABS, and could be affirmed by orderly lowering yield strength, as shown in the next section.

### 3.2. Effect of TiO_2_ with and without Calcination on Photoantibacterial Activity and Yield Strength of ABS

**Figure 3:**
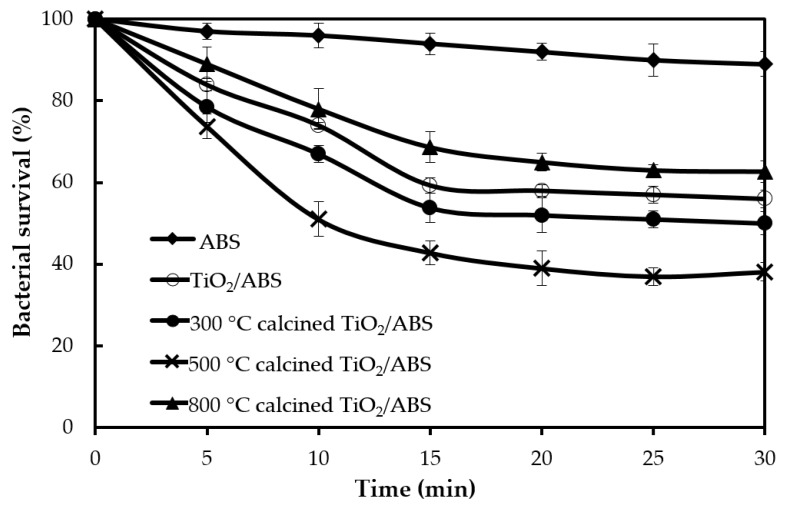
Bacterial survival following photocatalytic reaction of ABS, TiO_2_/ABS and 300–800 °C calcined TiO_2_/ABS (TiO_2_ concentration of 1 wt %).

**Figure 4:**
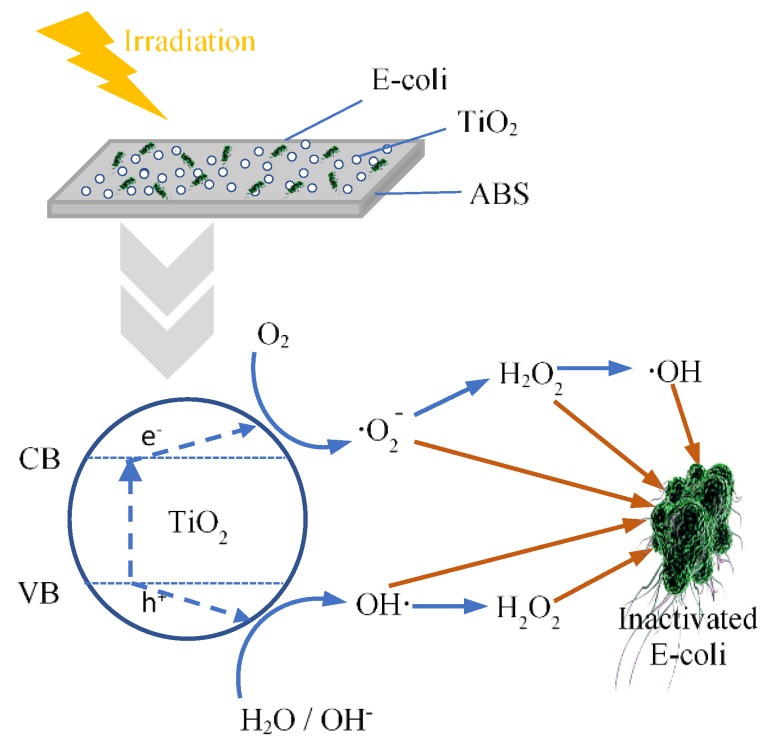
The mechanism underlying the photocatalytic antibacterial effect.

The influence of TiO_2_ and calcined TiO_2_ mixed with ABS on photoantibacterial *Escherichia coli* (*E. coli*) as assessed by the JIS Z 2801: 2010 standard test are illustrated in Figure 3. Photoantibacterial effectiveness for *E. coli* was presented in the form of bacterial survival. The result obtained for pure ABS under UV illumination for 30 min showed little antibacterial activity, while the *E. coli* survival of 60 ± 2.8% was observed in ABS with TiO_2_, highlighting the dominant impact of TiO_2_ in influencing photocatalytic activity. Figure 4 illustrates the mechanism of photocatalytic antibacterial action proposed, in which the OH radicals and reactive oxygen species (ROS) generated by TiO_2_ would damage the cell membrane, resulting in the leakage of bacterial cytoplasm, leading to cell death [20,21,22]. Moreover, the calcined TiO_2_ mixed with ABS provided a greater reduction of *E. coli* compared to uncalcined TiO_2_/ABS. Calcining TiO_2_ at 300 °C could decrease *E. coli* on ABS by 45 ± 2.1% (remaining bacterial survival 55%), further decreasing to 60 ± 2.6% at 500°C (remaining bacterial survival 40%), despite a decrease in surface area. The photocatalytic improvement could be plausibly explained by a higher degree of crystallinity as mentioned earlier. That is, the more-active crystal phase was improved and surface defects were reduced as documented by several studies [23,24,25,26]. However, when increasing calcination temperature up to 800°C, the photoantibacterial performance decreased. This may derive from sintering and agglomeration effects during calcination at high temperature, as shown in SEM imagery (Figure 2).

**Figure 5:**
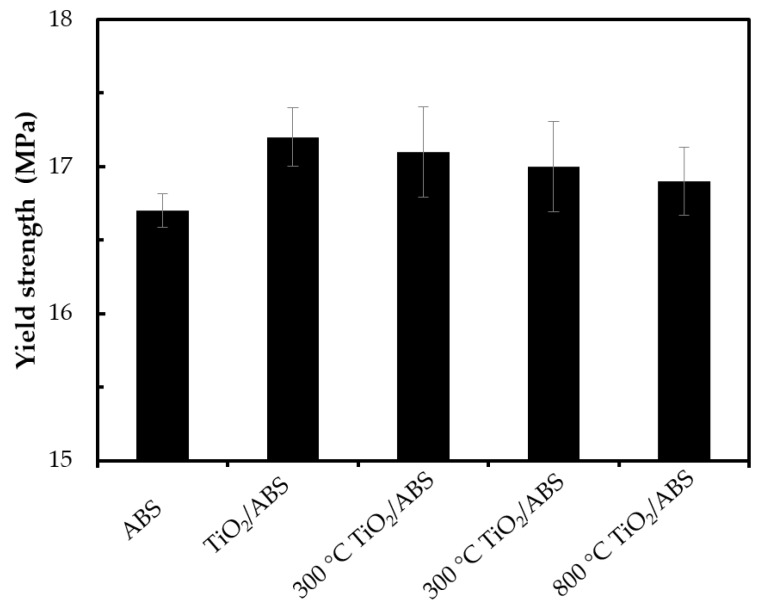
Yield strength of ABS, TiO_2_/ABS and 300 °C–800 °C-calcined TiO_2_/ABS (TiO_2_ concentration of 1 wt %).

Figure 5 shows that the yield strength of plain ABS was 16.7 ± 0.1 MPa, which was in the standard range of mechanical strength for this material. ABS containing TiO_2_ could further improve the yield strength of the workpiece. The enhancement of strength can be explained by TiO_2_ creating temporary crosslinks among the polymer chains during the deformation process. In particular, uncalcined TiO_2_/ABS showed the highest values of yield strength with a yield point of 17.2 ± 0.2 MPa. A slight decrease of yield strength was observed when increasing calcination temperature of TiO_2_. The yield strengths of 300 °C TiO_2_/ABS, 500 °C TiO_2_/ABS and 800 °C TiO_2_/ABS were 17.1 ± 0.3, 17.0 ± 0.3 and 16.9 ± 0.2 MPa, respectively. The decrease of yield strength may be attributed to increasing agglomeration and reduced surface area of TiO_2_ (e.g., a lower interfacial area for bonding to the ABS polymer) with increasing calcination temperature, as seen from SEM imagery (Figure 2), which contributed to formation of fewer crosslinks among the polymer chains.

### 3.3. Effect of Concentration of Calcined TiO_2_ on Photoantibacterial Activity and Yield Strength of ABS

**Figure 6:**
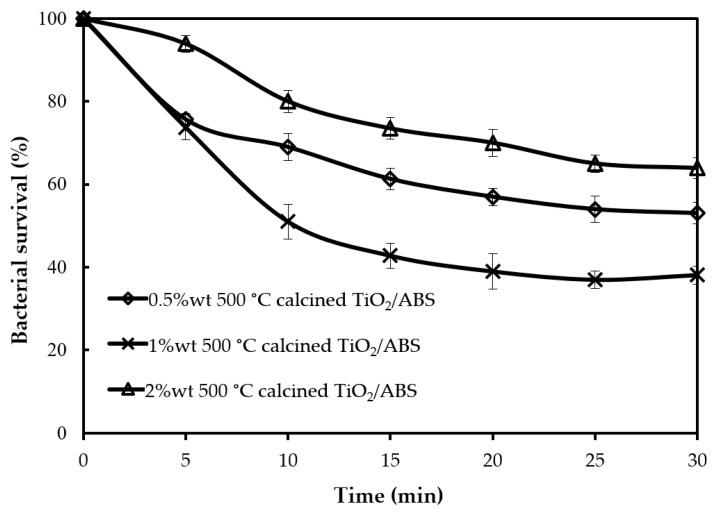
Bacterial survival by photocatalytic reaction of ABS with 500 °C calcined TiO_2_ at different concentrations.

The previous section demonstrates that optimum performance on photocatalytic performance occurred at 500 °C for calcined TiO_2_/ABS. In this section, the influence of 500 °C-calcined TiO_2_ concentration in ABS on photocatalytic performance was considered. From the 40% bacterial survival by 1 wt % calcined TiO_2_/ABS, changing of TiO_2_ concentration had been considered, as shown in Figure 6. The reduced concentration of TiO_2_ resulted in the increase of bacterial survival by 50 ± 2.2%. This indicated that the smaller amount of TiO_2_ was not enough to produce OH radicals and reactive oxygen species (ROS) to inactivate the bacteria. However, by increasing the concentration to 2 wt %, the bacterial survival increased to 70 ± 2.5%. This showed that presence of large amounts of TiO_2_ did not always lead to the high photocatalytic activity, but may in fact suppress the activity due to their aggregation.

**Figure 7:**
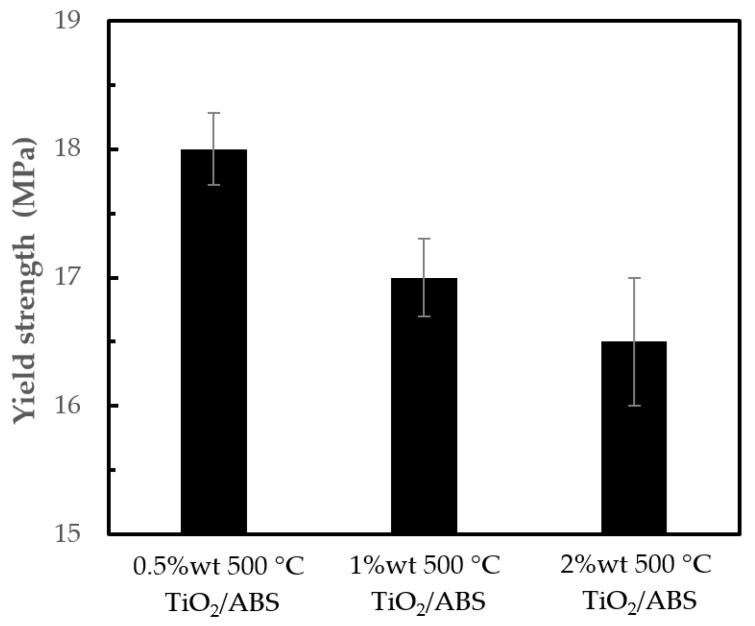
Yield strength of ABS with 500 °C calcined TiO_2_ at different concentrations.

Figure 7 shows that yield strength decreased with increasing of TiO_2_ loading. A 0.5 w t% TiO_2_/ABS had the highest yield strength of 18.0 ± 0.3 MPa. This was to be expected given the higher TiO_2_ inducing the effective matrix reduction. In other words, higher TiO_2_ loading (1 and 2 wt %) increase “particle-to-particle” interactions rather than “particle-to polymer” interactions, thus lowering yield strength [27]. 

### 3.4. Effect of Silane on Photoantibacterial Activity and Yield Strength of ABS

**Figure 8:**
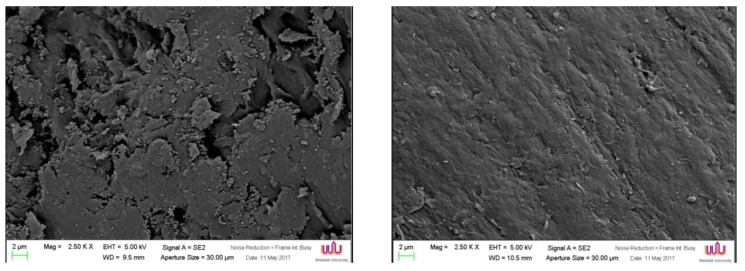
SEM of (**a**) 500 °C calcined TiO_2_/ABS and (**b**) 500 °C calcined TiO_2_/ABS with silane.

The influence of silane on TiO_2_/ABS was observed in the SEM images shown in Figure 8, in which the 500 °C-calcined TiO_2_/ABS without silane possessed rough surfaces and smaller particles on the ABS surface, as illustrated in Figure 8a. After mixing silane, the morphology of blended polymer in Figure 8b shows the presence of a smooth surface and better dispersion, rather than TiO_2_/ABS. The difference was attributed to a greater interactions with the polymer matrix, which silane coupling agent showed the compatible behaviour of ABS/TiO_2_ by creating more adherent bonding between TiO_2_ and ABS matrix, as similarly explained for the modified SiO_2_ with silane [28,29,30].

**Figure 9:**
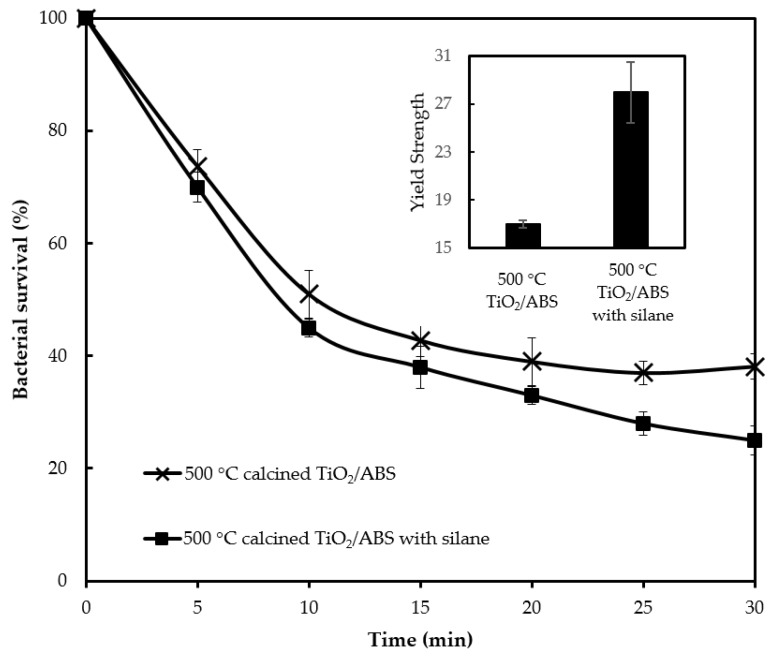
Bacterial survival by photocatalytic reaction of ABS with 500 °C calcined TiO_2_ (1 wt % loading) with and without silane and corresponding their yield strength (inset).

To investigate the effect of silane on photocatalytic activity, the ratio of TiO_2_ to silane during catalyst preparation was varied over the weight ratio of 1:0.2-1:0.5. The optimum photocatalytic activity occurred for samples at a ratio of TiO_2_ to silane of 1:0.3 as presented in Figure 9. Figure 9 reveals that 500 °C-calcined TiO_2_/ABS with silane (called “silane-TiO_2_/ABS” for brevity) displays a better photobacterial activity compared with 500 °C-calcined TiO_2_/ABS without silane. The silane-TiO_2_/ABS could reduce 75% of *E. coli* (remaining bacteria survival 25 ± 2.6%). The higher silane-TiO_2_/ABS photobacterial activity was attributed to the comparatively greater distribution of TiO_2_ on ABS which promoted good UV absorption; however this result did not agree with the findings of Pazokifard et al. [31], who reported that in a case of degradation of rhodamine, TiO_2_ P25 nanoparticles showed a better activity than silane-treated particles due to the reduced surface area of TiO_2_ affecting poor photon absorption. The differences between the photocatalytic activity findings for bacteria and rhodamine likely originate from the operating conditions when blending the composite and the particle itself. The inset of Figure 9 shows that the yield strength of silane-TiO_2_/ABS was 1.6 times higher than TiO_2_/ABS without silane. The yield strength improvement was ascribed to better dispersion and adhesion of TiO_2_ in the ABS matrix, which arose from the silane coupling agent, enhancing interfacial bonding between the TiO_2_ and the matrix. We could highlight one key finding here, that the incorporation of silane results in improvement of TiO_2_ particls dispersion within the ABS polymeric matrix (shown as higher photocatalytic activity) and increasing possible interactions between TiO_2_ particles and the matrix (shown as higher yield strength).

### 3.5. Reusability and Robustness

**Figure 10:**
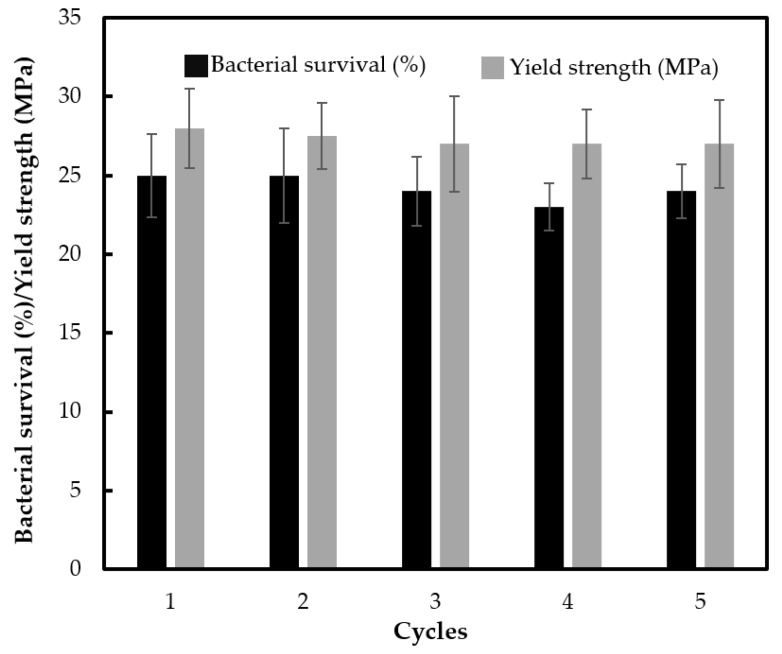
Reusability of ABS with 500 °C calcined TiO_2_ (1 wt % loading) with silane grafting up to the 5th cycle in terms of bacterial survival by photocatalytic reaction, and the yield strength of the material (photo irradiation time of 30 min for each cycle).

The stability and reusability in terms of photocatalytic activity and the robustness of the composite material are crucial for practical applications. Therefore, the antibacterial experiments were repeated without any treatment on the specimen between the cycle runs. Constant photocatalytic efficiency was observed after five reuse cycles without loss in the yield strength of material, as shown in Figure 10. This suggests that blending calcined TiO_2_ into ABS prevented the loss of TiO_2_ photocatalyst particles from the surface, which is usually observed when coating TiO_2_ on the polymer surface. As expected, Ti content in the ABS plastic after the fifth run was identical to the as-prepared material (as determined by inductively coupled plasma atomic emission spectroscopy (ICP-OES). This robust ABS material could minimize detachment and release of TiO_2_ leading to lessening of the possibly negative environmental fates, transport, transformation and toxicity.

## 4. Conclusions

Titanium dioxide (TiO_2_) was found to influence antibacterial performance and yield strength enhancement when blended with acrylonitrile-butadiene-styrene plastics (ABS). The optimum photoantibacterial activity occurred for ABS in the 500 °C-calcined TiO_2_ at a concentration of 1 wt %. At this temperature and concentration, the high degree of crystallinity and optimal amount of TiO_2_, were sufficient to produce OH radicals and reactive oxygen species (ROS), resulting in damage to bacterial cell membranes. The photoantibacterial performance for 500 °C calcined TiO_2_ at 1 wt % in ABS was more efficient than plain ABS over 62%. With optimal conditions, silane addition could further improve TiO_2_ dispersion on ABS. This resulted in a decrease of bacterial survival by 75%. Moreover, the benefit of TiO_2_-embedded ABS plastic could improve yield strength than pure ABS. The yield strength of TiO_2_/ABS with silane was 67.7% higher than of pure ABS. The efficiency of TiO_2_/ABS with silane photocatalyst showed an excellent photocatalytic antibacterial stability after five reuses, without loss in the yield strength.

## References

[1] Forrest S.R. (2004). The path to ubiquitous and low-cost organic electronic appliances on plastic. Nature.

[2] Saleh N.B., Milliron D.J., Aich N., Katz L.E., Liljestrand H.M., Kirisits M.J. (2016). Importance of doping, dopant distribution, and defects on electronic band structure alteration of metal oxide nanoparticles: Implications for reactive oxygen species. Sci. Total Environ..

[3] Plazas-Tuttle J., Das D., Sabaraya I.V., Saleh N.B. (2018). Harnessing the power of microwaves for inactivating Pseudomonas aeruginosa with nanohybrids. Environ. Sci. Nano.

[4] Hashimoto K., Irie H., Fujishima A. (2005). TiO_2_ photocatalysis: A historical overview and future prospects. JJAP.

[5] Gaya U.I., Abdullah A.H. (2008). Heterogeneous photocatalytic degradation of organic contaminants over titanium dioxide: A review of fundamentals, progress and problems. J. Photochem. Photobiol. C.

[6] Kiatkittipong K., Assabumrungrat S. (2017). A comparative study of sodium/hydrogen titanate nanotubes/nanoribbons on destruction of recalcitrant compounds and sedimentation. J. Clean. Prod..

[7] Lee S.L., Chang C.-J. (2019). Recent developments about conductive polymer based composite photocatalysts. Polymers.

[8] Mukherjee D., Barghi S., Ray A.K. (2014). Preparation and characterization of the TiO_2_ immobilized polymeric photocatalyst for degradation of aspirin under UV and solar light. Processes.

[9] Zhou T.-T., Zhao F.-H., Cui Y.-Q., Chen L.-X., Yan J.-S., Wang X.-X., Long Y.-Z. (2020). Flexible TiO_2_/PVDF/g-C3N4 Nanocomposite with Excellent Light Photocatalytic Performance. Polymers.

[10] Chawengkijwanich C., Hayata Y. (2008). Development of TiO_2_ powder-coated food packaging film and its ability to inactivate Escherichia coli in vitro and in actual tests. Int. J. Food Microbiol..

[11] Maneerat C., Hayata Y. (2006). Antifungal activity of TiO_2_ photocatalysis against Penicillium expansum in vitro and in fruit tests. Int. J. Food Microbiol..

[12] Kanazawa T., Ohmori A. (2005). Behavior of TiO_2_ coating formation on PET plate by plasma spraying and evaluation of coating’s photocatalytic activity. Surf. Coat. Tech..

[13] Ratova M., West G., Kelly P. (2014). Optimisation of HiPIMS photocatalytic titania coatings for low temperature deposition. Surf. Coat. Tech..

[14] Loddo V., Marcì G., Palmisano G., Yurdakal S., Brazzoli M., Garavaglia L., Palmisano L. (2012). Extruded expanded polystyrene sheets coated by TiO_2_ as new photocatalytic materials for foodstuffs packaging. Appl. Surf. Sci..

[15] Selvin T.P., Kuruvilla J., Sabu T. (2004). Mechanical properties of titanium dioxide-filled polystyrene microcomposites. Mater. Lett..

[16] Asiaban S., Taghinejad S.F. (2010). Investigation of the effect of Titanium Dioxide on optical aspects and physical and mechanical characteristics of ABS Polymer. J. Elastomers Plast..

[17] Sudeepan J., Kumar K., Barman T., Sahoo P. (2014). Tribological behavior of ABS/TiO_2_ polymer composite using Taguchi statistical analysis. Mater. Sci..

[18] Skorski M.R., Esenther J.M., Ahmed Z., Miller A.E., Hartings M.R. (2016). The chemical, mechanical, and physical properties of 3D printed materials composed of TiO_2_-ABS nanocomposites. Sci. Technol. Adv. Mater..

[19] Sangkatip R., Sriseubsai W., Kiatkittipong K. (2017). Antibacterial and Mechanical Properties of the TiO_2_/ABS Composites. Key Engineering Materials.

[20] Fagan R., McCormack D.E., Dionysiou D.D., Pillai S.C. (2016). A review of solar and visible light active TiO_2_ photocatalysis for treating bacteria, cyanotoxins and contaminants of emerging concern. Mat. Sci. Semicon. Proc..

[21] Ratova M., Mills A. (2015). Antibacterial titania-based photocatalytic extruded plastic films. J. Photoch. Photobio. A.

[22] Podporska-Carroll J., Panaitescu E., Quilty B., Wang L., Menon L., Pillai S.C. (2015). Antimicrobial properties of highly efficient photocatalytic TiO_2_ nanotubes. Appl. Catal. B.

[23] Yu J., Yu H., Cheng B., Trapalis C. (2006). Effects of calcination temperature on the microstructures and photocatalytic activity of titanate nanotubes. J. Mol. Catal. A Chem..

[24] An H., Zhu B., Li J., Zhou J., Wang S., Zhang S., Wu S., Huang W. (2008). Synthesis and characterization of thermally stable nanotubular TiO_2_ and its photocatalytic activity. J. Phys. Chem..

[25] Zhou W., Liu H., Wang J., Liu D., Du G., Cui J. (2010). Ag_2_O/TiO_2_ nanobelts heterostructure with enhanced ultraviolet and visible photocatalytic activity. ACS Appl. Mater. Interfaces.

[26] Yang H.G., Liu G., Qiao S.Z., Sun C.H., Jin Y.G., Smith S.C., Zou J., Cheng H.M., Lu G.Q. (2009). Solvothermal synthesis and photoreactivity of anatase TiO_2_ nanosheets with dominant {001} facets. J. Am. Chem. Soc..

[27] Rangari V., Reddy D.B. (2011). Polymer nanocomposite materials for structural applications. Advances in Nanocomposites-Synthesis, Characterization Industrial Applications.

[28] Li H., Zhang Z., Ma X., Hu M., Wang X., Fan P. (2007). Synthesis and characterization of epoxy resin modified with nano-SiO_2_ and γ-glycidoxypropyltrimethoxy silane. Surf. Coat. Technol..

[29] Xu X., Li B., Lu H., Zhang Z., Wang H. (2007). The interface structure of nano-SiO2/PA66 composites and its influence on material’s mechanical and thermal properties. Appl. Surf. Sci..

[30] Li X., Cao Z., Zhang Z., Dang H. (2006). Surface-modification in situ of nano-SiO_2_ and its structure and tribological properties. Appl. Surf. Sci..

[31] Pazokifard S., Mirabedini S., Esfandeh M., Mohseni M., Ranjbar Z. (2012). Silane grafting of TiO_2_ nanoparticles: Dispersibility and photoactivity in aqueous solutions. Surf. Interface Anal..

